# Correlating CpG islands, motifs, and sequence variants in human chromosome 21

**DOI:** 10.1186/1471-2164-12-S2-S10

**Published:** 2011-07-27

**Authors:** Leah Spontaneo, Nick Cercone

**Affiliations:** 1 Department of Computer Science and Engineering, York University Toronto, ON M3J 1P3, Canada

## Abstract

**Background:**

CpG islands are important regions in DNA. They usually appear at the 5’ end of genes containing GC-rich dinucleotides. When DNA methylation occurs, gene regulation is affected and it sometimes leads to carcinogenesis. We propose a new detection program using a hidden-markov model alongside the Viterbi algorithm.

**Methods:**

Our solution provides a graphical user interface not seen in many of the other CGI detection programs and we unify the detection and analysis under one program to allow researchers to scan a genetic sequence, detect the significant CGIs, and analyze the sequence once the scan is complete for any noteworthy findings.

**Results:**

Using human chromosome 21, we show that our algorithm finds a significant number of CGIs. Running an analysis on a dataset of promoters discovered that the characteristics of methylated and unmethylated CGIs are significantly different. Finally, we detected significantly different motifs between methylated and unmethylated CGI promoters using MEME and MAST.

**Conclusions:**

Developing this new tool for the community using powerful algorithms has shown that combining analysis with CGI detection will improve the continued research within the field of epigenetics.

## Background

Epigenetics studies the changes in gene function and gene expression that are not discernable by mutations in the DNA sequence. The area of biology devoted to epigenetics is a recent development and has a large amount of room for growth with new research on cancer, mammalian gene expression, and technological advances constantly being brought forth from the community. Epigenetic inheritance focuses on both mitotic and meiotic cellular changes and the processes involved. Looking at cell differentiation and genetic imprinting through epigenetics has created new leads for cancer research in terms of tumour growth. The chromatin that controls DNA processes is an epigenetic mechanism in either an active or repressive state. There are three main mechanisms in epigenetics: DNA methylation, histone modifications, and the binding of non-histone proteins [1].

CpG islands (CGIs) usually appear at the 5’ end of genes containing GC-rich dinucleotides. Normally, these regions are unmethylated; however, when methylation occurs, gene regulation is affected and methylation sometimes leads to carcinogenesis. The importance of CGIs has produced numerous algorithms throughout the community dedicated to locating and understanding these regions in DNA [2]. Many of the traditional algorithms use the measures of length, GC content, and the number of observed over expected CpGs when determining if a section of DNA is a CGI. However, some newer algorithms employ a distance based detection method to identify CpG clusters [3]. Some of the features of unmethylated CpGs are their affinity to bind to a protein domain (*CXXC3*), their low mutation rate, and their association with open chromatin. Low methylation frequency has been correlated with high CpG density and vice versa.

DNA methylation refers to the replacement of the attached hydrogen with a methyl group on a cytosine base, which causes the chromatin to become more compact affecting transcription factor binding [1]. Methylated cytosines will produce thymine when deaminated unlike unmethylated cytosines which produce uracil. CpG density is lost due to the thymine mutation and this often inhibits the promoter regions of genes [4]. The affect of methylation is particularly important in the case of cancer and the epigenetic modifications that silence tumour suppressor genes. It has been observed that genes silenced epigenetically share sequence motifs in their promoter regions. This is one of the possible ways to detect cancer hypermethylation [5].

In genetics, a sequence motif is a short pattern of nucleotides that is deemed to have biological significance. If a motif appears in the exonic region of a gene, it may encode the structural motif of a specific protein. Regulatory sequence motifs are located in areas where regulatory proteins such as transcription factors bind to the DNA. In order to find the most significant motifs in DNA, many algorithms have been designed and applied to different organisms [6]. When describing a motif, pattern notation using regular expressions is what depicts the sequence. The de novo computational methods to discover important motifs take multiple input sequences and try to generate candidate motifs. Two of the most well-known algorithms often used by many researchers are BLAST and MEME [7][8].

CpG islands are found in 40% of promoter and exonic regions of mammalian genes. Other areas of the genome contain very few CpG dinucleotides and these areas are normally methylated [9]. Methylation of promoter CpGs is known to cause gene silencing and is heavily implicated in carcinogenesis. Gardener-Garden and Frommer were the first researchers to use computational methods to detect and analyze CpG islands using specific criteria: 200-bp (base pair) length DNA region, GC content greater than 50%, and observed CpG/expected CpG ratio (Obs_CpG_/Exp_CpG_) greater than 0.6 [10]. Although the original criteria provided a good starting point for CpG island detection, it didn’t take into account repeating regions of DNA. Alu repeats are short interspersed elements repeating within the genome that are approximately 280-bp in length often containing a high GC content and Obs_CpG_/Exp_CpG_ ratio [11].

Takai and Jones analyzed human chromosomes 21 and 22 for CpG islands using their own algorithm that built upon the original criteria coined by Gardener-Garden and Frommer. The new algorithm reduced the number of detected CpG islands from 14,062 to 1,101, which is closer to the number of genes located on the two chromosomes (~750 genes). Using the same criteria, the new algorithm modifies the constraints of each criterion to produce better detection results. Now repeating elements such as Alu are not considered as often as before (from 7,651 to 122 Alus detected as CpG islands). The constraints for the new algorithm are as follows: length ≥ 500-bp, GC content ≥ 55%, and ObsCpG/ExpCpG ≥ 0.65.

CpGcluster [3] discovers clusters of CpGs by looking at the distance between other CpGs on the same chromosome and applying statistical significance. The two algorithms were compared using many different factors (length, GC content, ObsCpG/ExpCpG) to determine which is better at finding CGIs [2]. CpGcluster locates a much larger number of CGIs; however, after further analysis only 14.7% and 16.2% mapped to promoter regions of the human and mouse genomes respectively. The study found that often multiple clusters of CpGs from CpGcluster were substrings of one large CGI detected by the Takai and Jones algorithm. Since there is no length limitation when CpGcluster detects CGIs, a much larger number of clusters are discovered.

Gene expression is one of the most important functions in all forms of life. Transcription factors are encoded in about 3-5% of genes in eukaryotes allow the repression or activation of specific genes within DNA. Chromatin plays another significant role in gene regulation and the network of interactions between transcription factors and chromatin structure is becoming increasingly important in epigenetic research. DNA methylation is an epigenetic memory mechanism involved in the silencing of genes within eukaryotic organisms. Histone modifications are another instrument in the epigenetic inheritance that passes information from parent to daughter cells. Post-translational modifications of core histone proteins have been linked to transcription repression and activation [12].

Aberrant methylation of promoter regions of genes has been linked to gene silencing and loss of expression in diseases like cancer [1] and it is known that cancer mutation can cause alterations to protein signaling genes [13]. Single nucleotide polymorphisms (SNPs) are the most common variation in the genetic sequence of the human genome. Studies continue to provide an increasing amount of evidence that SNPs are correlated with cancer and can be used as indicators of the disease [14]. Analyzing sequence variants in motifs within promoter regions of methylated genes could provide significant disease markers and possible sites for therapeutic study.

In this study, we examine several problems related to CpG islands and DNA methylation of the promoter regions of genes:

• Design a method of CGI detection using powerful algorithms that improves performance while incorporating the ability to correlate the methylation status of the DNA with the location of the CGI.

• Incorporate the power of analysis alongside CGI detection for an all-in-one program that covers the needs of the community.

• Integrate motif finding into the detection algorithm, determine if the motifs are within CGIs, and verify if they are within a transcriptional start site (TSS).

The rest of the paper is organized as follows. We first present the conceptual framework on which the program was designed. Then the methodology of the finalized program is discussed. Finally, the first set of experiments is presented.

## Methods

The detection of CGIs has evolved since the first detection algorithm was proposed by Gardener-Garden and Frommer. Recently, studies have shown that incorporating the use of a hidden Markov model in a detection algorithm can improve results [15]. When establishing new techniques, it is still important to consider the traditional methods and integrate the best features of both into a new algorithm. In our work, we combined a hidden Markov model, the Baum-Welch algorithm, and the Viterbi algorithm along with the traditional sliding window criteria to lower the detection of repeating elements.

### Hidden markov model

A hidden Markov model (HMM) consists of a Markov process in which the state is unobservable. A Markov process is a random phenomenon where future probabilities are determined based on the most recent values. An HMM requires the knowledge of a few probabilities before it can be run on any data. These probabilities are split into three separate groups:

• initial probabilities - the probabilities that determine which state the system will be in during the start of the algorithm. Often, the initial probabilities are equal among the different states (i.e. 2 states: P(i) = 0.5, P(j) = 0.5).

• transition probabilities - the probabilities that provide the occurrence of a change from state i to state j (i.e. P(i|j) = 0.25, P(i|i) = 0.75).

• emission probabilities - the probabilities distinguishing each state based on the observations of the system (i.e. P(x|i) = 0.22, P(x|j) = 0.36).

### Estimating parameters

HMMs have three distinct sets of parameters or probabilities: the initial state probabilities that determine which state the system starts in; the transition probabilities that decide if the state will switch after a certain period of time; and the emission probabilities showing whether the current symbol output belongs to one state or another. For an HMM to decode a sequence into a path of states, the parameters need to be trained on a sequence of symbols to detect the final probabilities the system will use when finding the Viterbi path. One of the best and most efficient methods of estimating probabilities is through the use of the Baum-Welch algorithm, which is a special case of expectation-maximization.

The Baum-Welch algorithm is often used in HMMs to estimate the unknown parameters or probabilities. It is also known as the forward-backwards algorithm and is a special case of the generalized expectation-maximization algorithm. It can produce maximum likelihood and posterior mode estimates for model parameters when given only the emission probabilities to work with. The algorithm starts by assigning initial probabilities to all of the model parameters. Then it continues until convergence happens by adjusting the probabilities of each model parameter to increase the probability of the model in accordance with the training set being scanned.

### Viterbi algorithm

The Viterbi algorithm uses dynamic programming to find the most likely sequence that the hidden states would take based on the observations in a parameterized model. This sequence is called the Viterbi path and it is usually related to HMMs. The Viterbi algorithm is very similar to the forward algorithm which computes the probability that a set of observed events was generated by the model. The algorithm was designed in 1967 by Andrew Viterbi to decode convolutional codes within the noise of digital communication links.

The algorithm takes a HMM with possible *Q* states, initial probabilities *π_i_* where *i* is the current state of the model, and transition probabilities *a_i_*_,_*_j_* where *i*, *j* is the change from state *i* to state *j* . Given a sequence of observable data *x_0_*,…,*x_L_* , the algorithm will generate a state sequence *q_0_*, …,*q_L_* for each observable value. The algorithm produces the final output using recurrence relations.

*V_l,k_* is the probability of the most likely state sequence based on the current *l* + 1 observations. The state sequence can be recovered by saving in memory the state *q* is in during the run through the second equation. Then say there is a function, St(*k,l*) that returns the value of *q* which produced *V_l,k_* when *l* > 0 and *k* when *l* = 0. The Viterbi path can be discovered using the following:

### DNA methylation analysis

Once the CGI detection algorithm runs and scans the genetic sequence, the researcher can use the detected island locations to create primer sequences to determine the methylation status of the CGI. Often, a separate statistics program is used to calculate significance. In our work, the analysis of the data is available using the p-value derived from the Kolmogorov-Smirnov two-sample test and the distribution of methylated to unmethylated islands is tabulated through the calculation of the z-score. The Kolmogorov-Smirnov test uses minimum distance estimation to compare sample datasets with reference probability distributions equating them with a one-dimensional probability distribution. The test can be performed with one sample dataset (one-sample K-S test) or with two sample datasets (two-sample K-S test). The test either defines the mathematical distance between the empirical distribution function of a set of data and the cumulative distribution function of the reference distribution (one-sample) or the distance between the empirical distribution of two separate sets of data (two-sample). The samples calculated under the null hypothesis are taken from the reference distribution (one-sample) or the same distribution (two-sample) and form the null distribution for the test. When the Kolmogorov-Smirnov test is used as a goodness of fit test, the data is normalized and compared to a standard normal distribution.

The Kolmogorov-Smirnov statistic uses the empirical distribution function where  are a set of ordered data points,

where 1{*y_i_ ≤ x*} is the indicator function. The Kolmogorov statistic for a cumulative distribution function *E*(*x*) can be calculated using,

which calculates the supremum of the distances in the set, sup *x*. The Kolmogorov-Smirnov statistic often requires a large set of data to give an accurate acceptance or rejection of the null hypothesis; however, since we are working with the human genome and chromosomes seem to contain a large amount of CGIs [16][17] and thus our datasets should be large enough to produce accurate p-values. In our work, we use the two-sample K-S test to determine if two datasets (unmethylated and methylated CGIs) differ in their probability distributions in regards to length, GC content, and Obs/Exp ratio. The two-sample K-S test uses the Kolmogorov statistic

where  and  are the empirical distribution functions of each sample.

### Program architecture

There are a multitude of detection programs that use many different algorithms to accurately detect CGIs within a genetic sequence. Some of the most popular CGI detection algorithms are Gardener-Garden and Frommer [10], CpG Island Searcher [9], and CpGProd [18]. The Gardener-Garden and Frommer algorithm is the original CGI detection algorithm which uses a sliding window of 200 bp along with a GC content greater than 50% and an observed-to-expected CpG ratio greater than 0.6. The CpG Island Searcher built upon the original foundation using a window of 500 bp, GC content ≥ 55%, and Obs_CpG_/Exp_CpG_ ≥ 0.65. The CpGProd algorithm searches a genome for CGIs using two steps: (1) search for all CGIs in a submitted sequence based of the traditional criteria, and (2) predict the orientation of promoters once the start CGI is discovered [18].

We decided to design a graphical user interface (GUI) for our CGI detection program to provide accessibility for researchers that are not well-versed in scripting or working with programs depending on the command prompt. While both the CpG Island Searcher and CpGProd have a web server that provides a user interface, it is not feasible to run an dataset as large as a human chromosome through the web. Both programs must be run using typed commands and parameters if not running them through the web and do not provide the same interface as on the web for the user.

### Layout and design

**Figure 1:**
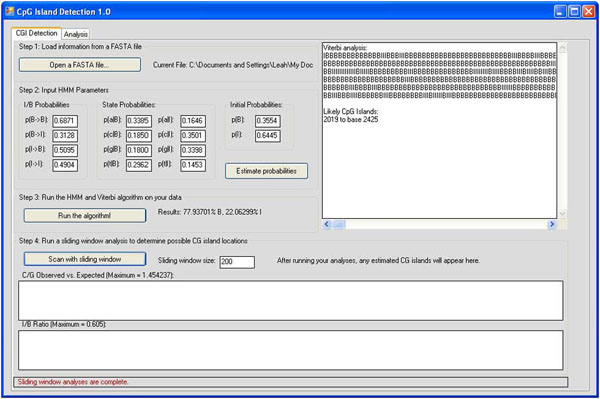
**CGI Detection 1.0 Layout**.

The original CGI detection layout and code was released by Tanner Helland (http://www.tannerhelland.com/) under the BSD license. It was originally written and designed in Microsoft Visual Basic 6.0, but we updated and modified the code using Microsoft Visual Basic .NET and Microsoft Visual Studio 2008. The GUI provides an intuitive method for loading the FASTA sequence file, setting up the HMM parameters, estimating the parameters based on the file, and running the algorithm. Once the Viterbi algorithm has defined when the sequence is in an island state ("I") or a normal state ("B"), the sliding window can be run to detect where the islands are located within the genomic sequence which show inside the graphs for Obs_CpG_/Exp_CpG_ ratio and I/B ratio. Once the sliding window has scanned for CGIs, the results are shown in the textbox to the right (Fig. 1).

We modified the layout to use a tab control in order to switch between the CGI detection and the analysis of the results. The analysis tab contains a small spreadsheet for the detected islands where the user can change the methylation status (methlyated/ unmethylated) and can enter a methylation score for each island. Methylation scores have been used in many different studies [16][19][20] as a measure of the strength of the methylation of a specific CGI to determine whether to classify it as methylated or unmethylated. Once the user has filled in the values for methylation status and score for each CGI, the analysis of the data can be completed.

**Figure 2:**
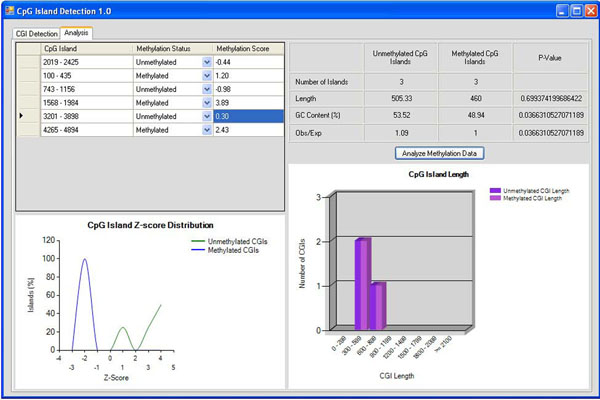
**CGI analysis layout and design**.

The table next to the spreadsheet displays the separate mean values for CGI length, GC content, and Obs_CpG_/Exp_CpG_ ratio for the unmethylated and methylated CGIs. The p-values are calculated using the Kolmogorov-Smirnov two-sample test. The chart underneath the spreadsheet displays the z-score distribution for the methylated and unmethylated CGIs determined by using the methylation scores entered by the user. The final chart shows the distribution of lengths of the CGIs across the two sets of data (Fig. 2).

We created this layout and design to provide practicality for users that are not computational experts and to offer an all-in-one solution for detecting and analyzing CGI data.

## Results

### Methylation analysis

**Figure 3:**
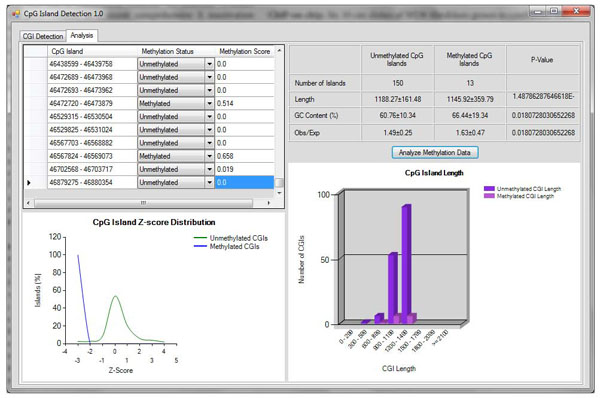
**Analysis of promoters found on chromosome 21**.

Looking at the composition of CGIs when methylated and unmethylated is important in the study of the epigenetic mechanism of methylation. The list of promoters from Weber et al. [21] was evaluated by taking the promoters found within chromosome 21 and analyzing them using the CpG Island Detection 1.0 program analysis tab. The 163 promoters were selected based on the promoter class given to each in the previous work. Those with a class of HCP or ICP were considered to contain CGIs within or covering the promoter region when looking at methylation. A CGI was considered methylated if the 5mC log2 ratio 0.4 and unmethylated otherwise. Using these criteria, the promoter regions were run through the program, using the 5mC log2 ratio for the methylation score of each island (Fig. 3).

Of the 163 promoters, 13 were considered methylated and 150 were unmethylated. This distribution is unsurprising considering that promoters which are methylated tend to silence the function associated with the gene. The p-values calculated by the Kolmogorov-Smirnov test clearly show the significance between methylated and unmethylated CGIs with all categories being notably less than 0.05 as shown in Table 1.

**Table 1:** Comparison of methylated and unmethylated promoter CGIs.

	Unmethylated CpG Islands	Methylated CpG Islands	P-Value
**Number of Islands**	150	13	
**Length**	1188.27±161.48	1145.92±359.79	< 1.48 x 10^-11^
**GC Content (%)**	60.76±10.34	66.44±19.34	< 1.81 x 10^-2^
**Obs_CpG_/Exp_CpG_**	1.49±0.25	1.63±0.47	< 1.81 x 10^-2^

Methylated and unmethylated promoter CGIs were compared by sequence for average length, GC content (%), and ObsCpG/ExpCpG ratio. P-values were determine using the Kolmogorov-Smirnov two-sample test.

The unmethylated islands show a normal distribution according to the z-scores; however, it is hard to discern any important findings from the methylated CGIs. This is possibly due to the lack of methylated islands in this dataset. A larger count of methylated CGIs might have provided some deeper insight into how they tend to be distributed throughout the genome. Looking at the allocation of island length, it is clear that most islands within promoters are somewhere in the range of 900 - 1500 bp long. With a larger set of methylated CGIs, there might have been a more informative conclusion as to whether longer islands, such as those in promoters, are more susceptible to methylation.

### CGI motifs

It is still widely unknown as to why certain CGIs are more susceptible to DNA methylation as compared to others. The possibility that CGIs, especially those located within promoter regions and covering TSSs, contain motifs that are more likely to mutate or allow methylation has not yet been thoroughly researched. To determine if any of the CGIs contained similar motifs, MEME was run on 5 of the total 13 methylated promoters to discover the top 3 most significant methylated sequence motifs. The motifs were then checked against the entire 13 CGIs by using the MAST tool for alignment. A similar experiment was performed for 13 randomly chosen unmethylated promoter CGIs. It is interesting to note that even from this small sample of CGIs, a large percentage (76.9%) of methylated islands contain the top motif; whereas, less than half of the unmethylated islands contain that same motif (38.4%). The third motif for methylated CGIs contains the most surprising result showing that it was almost non-existant within the scanned unmethylated CGIs as compared to the methylated promoters (7.7% and 61.5% respectively).

However, when looking at the unmethylated island motifs, both the methylated and unmethylated CGIs have almost equal occurrences for the top 3 hits. With a larger database of methylated CGIs, a more significant result could be obtained in terms of the motifs found within the islands, especially the CGIs that cover promoter regions and TSSs.

## Discussion

In our work we designed a brand new CGI detection tool, CpG Island Detection 1.0. It uses a hidden-Markov model and the Viterbi algorithm to detect CGIs within mammalian genomes. The parameters of the model are estimated using the Baum-Welch algorithm and the final method of locating islands uses a sliding window of a size specified by the program user. The tool provides a graphical user interface for users, allowing textbox entry and one click results. Even with the limitations in computational power, CpG Island Detection 1.0 stood up well against the Takai and Jones CpG Island Searcher. It was able to detect 347 CGIs within human chromosome 21 and the average lengths and Obs_CpG_/Exp_CpG_ ratios were in line with that of the Takai and Jones method, 1206 bp and 0.87 respectively.

A list of 163 promoters within chromosome 21 from Weber et al. was analyzed using the tool's analysis tab. The methylation status was entered from the original data and after the tool's analysis some significant results were obtained. There is a definite difference between methylated and unmethylated islands and the compositions of their characteristics (Length, GC content, Obs_CpG_/Exp_CpG_ ratio) which all had p-values less than 0.05 (as indicated by the Kolmogorov-Smirnov two-sample test). The z-score graph produced by the dataset showed that unmethylated CGIs show a normal distribution as well. A larger dataset of methylated islands is required in order to see if there is anything interesting in regards to how they are distributed.

Finally, the 13 methylated promoter regions and another 13 unmethylated regions were run through MEME and MAST to determine if there are any significant motifs shared between CGIs. The methylated islands shared the 3 top motifs with percentages of 76.9%, 69.2%, and 61.5% respectively; whereas, those motifs were only found in less than 50% of the unmethylated CGIs. The motifs found within the unmethylated islands were equally distributed throughout both the methylated and unmethylated regions. This indicates a possible correlation between motifs and methylation in regards to gene silencing. Those genes more likely to become methylated may contain motifs prone to methylation and mutation.

## Conclusions

Using an entire genome analysis will provide better insight into the analysis of methylated and unmethylated islands. A more recognizable distribution for methylated islands might be possible with a larger set of data points to consider. Acquiring more data will better showcase the power of the analysis tool and the assistance it provides to users scanning genomic regions for CGIs. The tool will be improved to allow a user to save their results and export the located CGIs to a file for other experiments and analyses. It will also be upgraded to allow a user to import located CGIs from a previous session or file, rather than requiring a rescan of a sequence every time an analysis needs to be completed.

Obtaining a larger number of methylated islands for analysis with MEME and MAST will provide more significant results in terms of motifs within CGIs. Looking across the entire genome and the promoter regions found within a multitude of chromosomes could show whether the methylation-prone motifs are consistent within susceptible CGIs across the genome and provide an insight into why certain genes become silenced within diseases such as cancer. Developing this new tool for the community using powerful algorithms has shown that combining analysis with CGI detection will improve the continued research within the field of epigenetics.

## Competing interests

The author(s) declare that they have no competing interests.

## Authors' contributions

LS created the program and carried out the experiments. NC provided advice and resources to perform all of the required tests and design the algorithm and helped draft the manuscript. All authors read and approved the final manuscript.

## References

[B1] Bock C Lengauer T Computational epigenetics Bioinformatics 2008 24 1 1 10 10.1093/bioinformatics/btm546 18024971

[B2] Zhao Z Han L Cpg islands: algorithms and applications in methylation studies Biochemical and biophysical research communications 2009 382 4 643 645 10.1016/j.bbrc.2009.03.076 19302978 PMC2679166

[B3] Hackenberg M Previti C Luque-Escamilla PL Carpena P Martinez-Aroza J Oliver JL Cpg cluster: A distance-based algorithm for cpg-island detection BMC Bioinformatics 2006 7 446+ 10.1186/1471-2105-7-446 17038168 PMC1617122

[B4] Siegfried Z Simon I DNA methylation and gene expression Wiley Interdisciplinary Reviews: Systems Biology and Medicine 2009 2 362 371 10.1002/wsbm.64 20836034

[B5] Goh L Murphy SK Muhkerjee S Furey TS Genomic sweeping for hypermethylated genes Bioinformatics 2007 23 3 281 288 10.1093/bioinformatics/btl620 17148511

[B6] Das MK Dai HKK A survey of dna motif finding algorithms BMC bioinformatics 2007 8 Suppl 7 S21+ 18047721 10.1186/1471-2105-8-S7-S21 PMC2099490

[B7] Li N Tompa M Analysis of computational approaches for motif discovery Algorithms for molecular biology : AMB 2006 1 1 8+ 10.1186/1748-7188-1-8 16722558 PMC1540429

[B8] McGinnis S Madden TL)Blast: at the core of a powerful and diverse set of sequence analysis tools Nucleic Acids Res 2004 32 Web Server issue 10.1093/nar/gkh435 PMC441573 15215342

[B9] Takai D Jones PA Comprehensive analysis of cpg islands in human chromosomes 21 and 22 Proc Natl Acad Sci U S A 2002 99 6 3740 3745 10.1073/pnas.052410099 11891299 PMC122594

[B10] Gardiner-Garden M Frommer M Cpg islands in vertebrate genomes Journal of molecular biology 1987 196 2 261 282 10.1016/0022-2836(87)90689-9 3656447

[B11] Schmid CW Does sine evolution preclude alu function? Nucl. Acids Res 1998 26 20 4541 4550 10.1093/nar/26.20.4541 9753719 PMC147893

[B12] van Steensel B Mapping of genetic and epigenetic regulatory networks using microarrays Nature Genetics 2005 37 Suppl S18 S24 15920525 10.1038/ng1559

[B13] Bianco R Melisi D Ciardiello F Tortora G Key cancer cell signal transduction pathways as therapeutic targets European journal of cancer 2006 42 3 290 294 10.1016/j.ejca.2005.07.034 16376541

[B14] Bond GL Hu W Levine A A single nucleotide polymorphism in the MDM2 gene: from a molecular and cellular explanation to clinical effect Cancer Res 2005 65 5481 5484 10.1158/0008-5472.CAN-05-0825 15994915

[B15] Wu H Caffo B Jaffee HA Irizarry RA Feinberg AP Redefining cpg islands using hidden markov models Biostatistics 2010 11 3 499 514 10.1093/biostatistics/kxq005 20212320 PMC2883304

[B16] Straussman R Nejman D Roberts D Steinfeld I Blum B Benvenisty N Simon I Yakhini Z Cedar H Developmental programming of cpg island methylation profiles in the human genome Nature structural & molecular biology 2009 16 5 564 571 10.1038/nsmb.1594 19377480

[B17] Yamada Y Shirakawa T Taylor TD Okamura K Soejima H Uchiyama M Iwasaka T Mukai T Muramoto K Sakaki Y Ito T A comprehensive analysis of allelic methylation status of cpg islands on human chromosome 11q: comparison with chromosome 21q DNA sequence : the journal of DNA sequencing and mapping 2006 17 4 300 306 10.1080/10425170600886128 17312950

[B18] Ponger L Mouchiroud D CpGProd: identifying CpG islands associated with transcription start sites in large genomic mammalian sequences Bioinformatics 2002 18 631 633 10.1093/bioinformatics/18.4.631 12016061

[B19] Dai W Teodoridis JM Graham J Zeller C Huang THM Yan P Vass KJ Brown R Paul J Methylation linear discriminant analysis (mlda) for identifying differentially methylated cpg islands BMC Bioinformatics 2008 9 337+ 10.1186/1471-2105-9-337 18691414 PMC2529322

[B20] Lapidus RG Nass SJ Butash KA Parl FF Weitzman SA Graff JG Herman JG Davidson NE Mapping of ER Gene CpG Island Methylation by Methylation-specific Polymerase Chain Reaction Cancer Res 1998 58 2515 2519 9635570

[B21] Bird AP Cpg islands as gene markers in the vertebrate nucleus Trends in Genetics 1987 3 342 347

[B22] Weber M Hellmann I Stadler MB Ramos L Pääbo S Rebhan M Schübeler D Distribution, silencing potential and evolutionary impact of promoter dna methylation in the human genome Nature genetics 2007 39 4 457 466 10.1038/ng1990 17334365

